# Clinical, Echocardiographic, and Procedural Characteristics of Eccentric Versus Circumferential Pericardial Effusions in a Symptomatic Drainage Cohort

**DOI:** 10.31083/RCM48796

**Published:** 2026-08-19

**Authors:** Dacheng Fu, Ling Liu, Leilei Guo, Guangxia Wang, Guoshu Wang, Chunchang Qin

**Affiliations:** ^1^Department of Cardiovascular Medicine, Cardiovascular Research Center, The First Affiliated Hospital of Chongqing Medical University, 400042 Chongqing, China; ^2^Department of Cardiology, Jiangyou People’s Hospital, 621000 Mianyang, Sichuan, China; ^3^Department of Cardiology, Yibin Second People’s Hospital, 644000 Yibin, Sichuan, China; ^4^Department of Radiology, The First Affiliated Hospital of Chongqing Medical University, 400042 Chongqing, China

**Keywords:** pericardial effusion, echocardiography, pericardiocentesis, fluoroscopy, cardiac tamponade

## Abstract

**Background:**

Eccentric pericardial effusions are non-circumferential fluid collections that may complicate conventional pericardiocentesis, yet the associated clinical characteristics remain poorly defined.

**Methods:**

This single-center retrospective comparative cohort study included adult patients with symptomatic pericardial effusion of undetermined etiology who underwent percutaneous pericardiocentesis under transthoracic echocardiographic or fluoroscopic guidance or a surgical pericardial window procedure. Based on preprocedural multi-view transthoracic echocardiography (TTE), effusions were classified as eccentric or circumferential. Baseline characteristics, imaging features, procedural outcomes, pericardial fluid analyses, etiologies, and short-term outcomes were compared. Univariable and multivariable logistic regression analyses were performed to identify factors associated with eccentric distribution.

**Results:**

Among 156 patients, 47 (30.1%) had eccentric pericardial effusions. In multivariable analysis, a chronic course (odds ratio (OR) 6.31, 95% confidence interval (CI) 2.68–14.85; *p* < 0.001) and higher body temperature (per 1 °C increase: OR 2.52, 95% CI 1.13–5.63; *p* = 0.024) were independently associated with eccentric effusion. Compared with circumferential effusions, eccentric effusions showed a different distribution of dominant maximal-depth sites (overall *p* = 0.026), with a higher proportion of maximal depth located along the left ventricular lateral wall (57.45% vs. 33.03%). Eccentric effusions also had a shallower anterior echo-free space (5 [3–7] vs. 11 [7–15.9] mm, *p* < 0.001), and showed fewer classic tamponade signs, including right ventricular diastolic collapse (12.77% vs. 39.45%, *p* < 0.001). Procedurally, eccentric effusions more frequently required fluoroscopic guidance (44.68% vs. 10.09%, *p* < 0.001), had lower drainage volumes (600 mL vs. 914 mL, *p* = 0.005), and showed a higher incidence of poor drainage (44.68% vs. 8.26%, *p* < 0.001). The etiologic spectrum of eccentric effusions was more heterogeneous and less likely to be malignant (31.91% vs. 55.96%). In-hospital and 90-day outcomes were comparable between groups.

**Conclusions:**

In this symptomatic drainage cohort, eccentric pericardial effusions were associated with a chronic course and higher body temperature and were characterized by left ventricular lateral predominance, fewer classic echocardiographic signs of tamponade, and greater reliance on fluoroscopic guidance for drainage. The etiologic spectrum of eccentric effusions was more heterogeneous and less likely to be malignant.

## 1. Introduction

Symptomatic pericardial effusions represent a common clinical manifestation shared by a wide spectrum of diseases, including malignancy, infection, metabolic disorders, autoimmune conditions, and iatrogenic causes [1]. It typically presents with dyspnea or chest discomfort and may progress to cardiac tamponade, necessitating prompt evaluation and intervention. In clinical practice, even after standardized diagnostic work-up and follow-up, the etiology remains indeterminate in up to 30% of clinically significant pericardial effusions [2,3]. Pericardiocentesis, which allows for the direct analysis of pericardial fluid for biochemical, microbiological, and cytological markers, provides valuable diagnostic information, particularly in distinguishing between malignant and infectious causes [4]. However, in some patients, the eccentric distribution of the pericardial effusion may hinder percutaneous drainage, limiting access to fluid for etiologic evaluation.

Under normal circumstances, small pericardial effusions initially accumulate posterior to the left ventricle and become circumferential as the volume increases [5]. Nevertheless, a subset of patients exhibits asymmetric, localized, or septated collections—referred to as eccentric or loculated effusions—even when the overall effusion volume is moderate to large. Previous guidelines and reviews have broadly classified pericardial effusions by spatial distribution into circumferential and loculated types [1,4,5,6], and in some studies, the terms loculated and eccentric were used interchangeably [7,8,9]. A loculated effusion implies focal fluid accumulation caused by adhesions or fibrotic septations. However, in clinical practice, non-circumferential, laterally localized effusions can also be observed in the absence of clear septations [8,9,10,11]. The continued use of the term “loculated” in such cases may misleadingly imply a fibrotic or post-inflammatory etiology. To avoid this mechanistic presupposition and to emphasize the anatomical distribution pattern, the term “eccentric” may be more appropriately used to describe non-circumferential, uneven pericardial fluid accumulation, particularly in the absence of definitive septations. Importantly, this eccentric distribution often increases the procedural complexity of percutaneous pericardial drainage.

For circumferential effusions, when the anterior echo-free space exceeds 10 mm, transthoracic echocardiography (TTE) provides a safe and effective route for pericardiocentesis and remains the most widely used imaging guidance technique in clinical practice [4]. In contrast, eccentric effusions—particularly those posteriorly or localized laterally—often lack a safe acoustic window for needle access under TTE alone, resulting in procedural challenges [10,11,12]. In such scenarios, fluoroscopic guidance offers a valuable alternative by enabling real-time lateral visualization of the needle trajectory and confirmation of the pericardial space using a minimal contrast injection, thereby reducing the risk of myocardial or pleural injury and improving both safety and procedural success rates [12,13,14,15].

In previous studies, eccentric or loculated pericardial effusions have been most frequently reported in postoperative settings, most likely reflecting localized injury or adhesions following cardiac surgery [16]. The etiology in such cases is typically clear. However, in non-iatrogenic patients with undiagnosed pericardial effusion, systematic comparative studies of eccentric and circumferential morphologies, particularly regarding clinical characteristics, imaging features, pericardial fluid profiles, and prognostic outcomes remain limited.

Therefore, we conducted a single-center retrospective study enrolling consecutive patients with a symptomatic, undiagnosed pericardial effusion who underwent percutaneous pericardiocentesis (TTE or fluoroscopic guidance) or surgical pericardial window. Patients with eccentric distribution were compared with circumferential cases from the same period. We aimed to compare the baseline clinical characteristics, echocardiographic phenotypes, pericardial fluid features, etiologic composition, and short-term outcomes between the two groups, and to explore the clinical implications of an eccentric pericardial effusion.

## 2. Materials and Methods

### 2.1 Study Design and Population

**Fig. 1.**
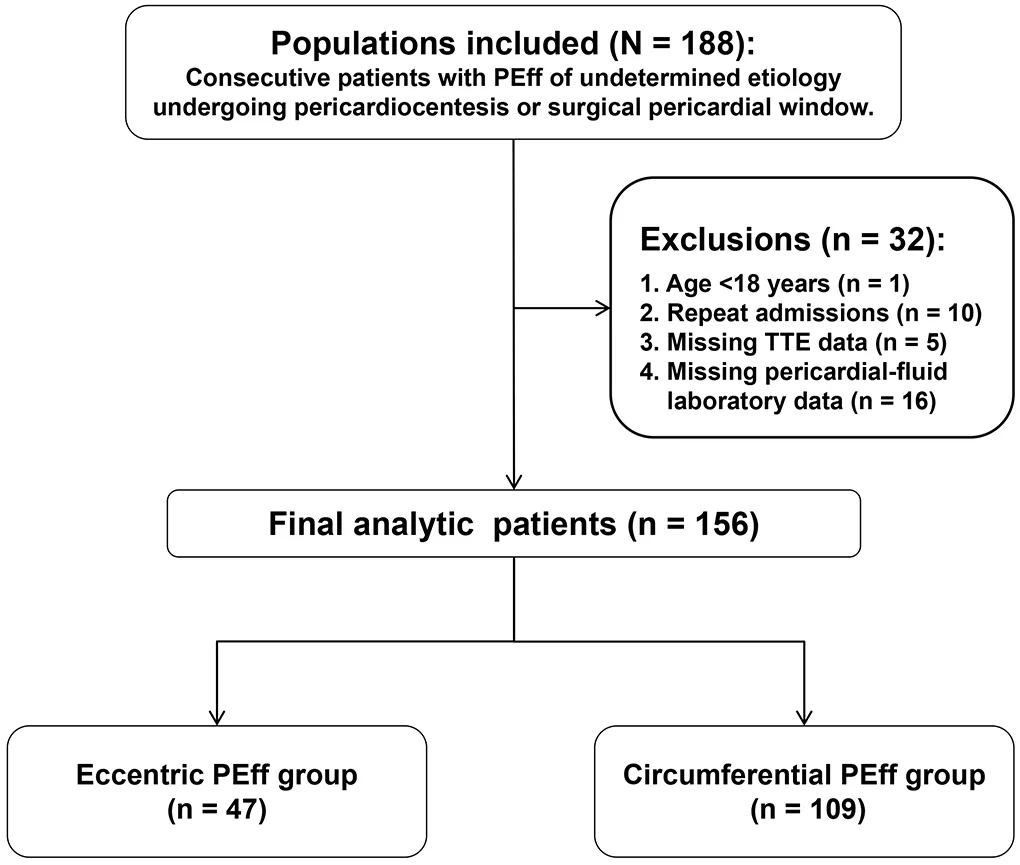
**The flow chart of the enrolled patients throughout the study**. PEff, pericardial effusion; TTE, transthoracic echocardiography.

This single-center retrospective comparative cohort study was conducted at the First Affiliated Hospital of Chongqing Medical University, and reported in accordance with the STROBE checklist for cohort studies (**Supplementary Table 1**). As illustrated in Fig. 1, we consecutively enrolled 188 hospitalized patients between January 2021 and July 2025 who presented with a symptomatic pericardial effusion of undetermined etiology and underwent either pericardiocentesis or surgical pericardial window. Exclusion criteria were as follows: (1) age <18 years (n = 1); (2) repeated hospital admissions (n = 10); (3) absence of essential echocardiographic data (n = 5); and (4) missing pericardial fluid laboratory results (n = 16). After applying these criteria, 156 patients were included in the final analysis. These patients were subsequently classified into two groups according to the anatomical distribution of the pericardial effusion: the eccentric group and the circumferential group.

### 2.2 Grouping Criteria

The distribution pattern of pericardial effusions in this study was determined using pre-procedural multi-view TTE. In accordance with our institutional protocol, each patient underwent a comprehensive TTE examination before pericardiocentesis or surgical intervention, including parasternal long-axis (PLAX), parasternal short-axis (PSAX), apical four-chamber (A4C), and subcostal views. These standard imaging planes were used to evaluate both the volume and spatial distribution of the effusion [17]. During the examination, the maximal echo-free space (EFS) was measured in four directions: anterior (external to the right ventricular anterior wall), posterior (external to the left ventricular posterior wall), left lateral (external to the left ventricular lateral wall), and right lateral (external to the right ventricular lateral wall). These measurements were then used to classify the distribution of the effusion and to guide planning for the trajectory of the pericardiocentesis. Because the study population consisted of symptomatic patients who underwent pericardiocentesis or surgical pericardial window for etiologic evaluation, the cohort contained no small effusions. Therefore, this classification framework was developed for moderate-to-large effusions in a clinical setting where drainage is performed.

Based on these criteria, a circumferential effusion was defined as a concentric fluid collection surrounding the heart in multiple echocardiographic views. An eccentric effusion was defined as a non-circumferential, laterally localized accumulation confined to one or two adjacent cardiac surfaces—anterior, posterior, or lateral—where the dominant echo-free space was at least twice the depth of the contralateral region. The 2:1 threshold was selected as a pragmatic operational cut-off to capture clearly asymmetric, clinically relevant non-circumferential effusions while avoiding classification of mild or borderline unevenness as eccentric, because no validated quantitative echocardiographic standard is currently available for this purpose. The robustness of this definition was further assessed in supplementary sensitivity analyses (**Supplementary Tables 2,3**).

### 2.3 Selection and Procedure of Pericardiocentesis

The selection of the puncture method was primarily determined by the anatomical distribution of the pericardial effusion. For patients with an anterior effusion (external to the right ventricular anterior wall) with a depth measuring greater than 10 mm, conventional bedside TTE-guided pericardiocentesis was performed. Common access routes included the subxiphoid, apical, or parasternal approaches. After puncturing into the pericardial cavity, aspiration of pericardial fluid was used to confirm entry, followed by placement of a drainage catheter for continuous drainage.

In contrast, for patients with an anterior effusion depth <10 mm, posterior or lateral effusions, or loculated effusions inaccessible via the standard transthoracic route, pericardiocentesis was performed under fluoroscopic guidance in the catheterization laboratory. The procedure followed the “CQMU pericardiocentesis technique” as previously described [15]. Under a left lateral fluoroscopic view, a subcutaneous puncture was made via the left costophrenic angle using an 18-gauge needle equipped with a blunt-tip metal cannula, with the instrument angled approximately 30° toward the left shoulder and directed toward Larrey’s triangle. A 0.014-inch Sion guidewire (stiff end) was advanced tangentially along the myocardial surface to penetrate the parietal pericardium and enter the pericardial space, after which a 1.7 Fr microcatheter was inserted and exchanged for the soft end of the guidewire. A small amount of contrast medium was injected when necessary to confirm intrapericardial positioning. For patients with pericardial adhesions, an optimized “guidewire probing and adjustment” strategy using repeated soft-tip manipulation was employed to facilitate catheter placement and maintain continuous drainage. This technique has been validated in both animal and clinical studies, demonstrating safety and efficacy in patients with dry or small anterior effusions and pericardial adhesions [15]. In a small subset of patients with circumferential effusions in whom bedside puncture was deemed high risk, pericardiocentesis under fluoroscopic guidance in the catheterization laboratory was also utilized to enhance procedural safety.

When percutaneous access was not feasible or had failed, patients were referred to cardiothoracic surgery to perform a surgical pericardial window.

### 2.4 Definition of Key Variables and Outcomes

Baseline variables were defined as information available prior to pericardiocentesis or surgery. Presenting symptoms (chest pain, dyspnea, and fever) and vital signs were also recorded. Laboratory parameters were obtained before the procedure.

Echocardiographic indices derived from pre-procedural TTE included maximal effusion depth, distribution, presence of septations, pericardial thickening or echogenicity, right ventricular (RV) diastolic collapse, and swinging heart. Procedure-related variables included guidance modality (TTE or fluoroscopy-guided), duration of catheter drainage, and total volume drainage. Pericardial fluid characteristics were recorded, including gross appearance, cytological cell counts and differentiation, protein, lactate dehydrogenase (LDH), adenosine deaminase (ADA), microbiological testing, and cytopathological examination.

Pericardial effusions were categorized according to course of the disease as acute (<1 week), subacute (1 week to 3 months), or chronic (>3 months) [4]. Etiological classification followed previously established guidelines [1,2]. (1) Malignant: Pericardial effusions were classified as definite malignant when malignant cells were identified on pericardial fluid cytology or tissue pathology. In the absence of positive cytology/pathology, effusions were classified as presumed malignant only when the patient had an active known malignancy together with imaging or clinical evidence suggesting pericardial involvement, and no other plausible alternative etiology was identified. (2) Tuberculous: Defined as definite when Mycobacterium tuberculosis was identified in the pericardial fluid or tissue by smear, culture, or histology; or probable when pericarditis occurred with tuberculosis elsewhere and responds to anti-tuberculous therapy [18]. (3) Chronic kidney disease (CKD)-related: Classified when the pericardial effusion occurred in patients meeting diagnostic criteria for CKD (structural or functional renal abnormality ≥3 months, or estimated glomerular filtration rate (eGFR) <60 mL/min/1.73 m^2^ for ≥3 months) after excluding other reasonable causes. (4) Hypothyroidism-related: Defined as a pericardial effusion associated with confirmed primary or secondary hypothyroidism (elevated thyroid-stimulating hormone (TSH) with decreased free thyroxine (FT4), or documented prior diagnosis/treatment), typically characterized by gradual development of serous effusion after exclusion of alternative etiologies. (5) Autoimmune: Included patients with systemic autoimmune diseases who had clinical or immunological evidence of disease activity consistent with pericardial involvement and without more plausible alternative causes. (6) Post-myocardial infarction (Post-MI) syndrome: Defined as part of the post-cardiac injury syndrome, occurring several weeks after myocardial infarction and characterized by pericarditis or an effusion accompanied by chest pain, low-grade fever, elevated C-reactive protein (CRP), and pericardial fluid accumulation. (7) Heart failure: Attributed to hemodynamic transudation secondary to decompensated heart failure or pulmonary hypertension, typically presenting as a small-to-moderate serous or transudative effusion that improved with diuresis or hemodynamic optimization. (8) Idiopathic: Diagnosed when no specific etiology could be identified after comprehensive evaluation.

Cardiac tamponade was defined by the presence of pericardial effusion on echocardiography, appearing as an anechoic or hypoechogenic space surrounding the heart, combined with clinical signs of hemodynamic instability [19]. Major complications included myocardial or coronary injury, hemorrhage >200 mL, clinically significant arrhythmia requiring treatment, infection, or pneumothorax. Poor drainage was defined as continuous drainage <50 mL within 24 hours, accompanied by a residual effusion ≥10 mm on repeat echocardiography or requiring catheter repositioning. Effusion recurrence was defined as re-accumulation of pericardial fluid with an echo-free space >10 mm within 90 days after discharge. Follow-up data on effusion recurrence and reintervention within 90 days were obtained from outpatient records and telephone interviews. To improve data reliability, the extracted data were independently cross-checked by two researchers, thereby reducing the risk of subjective deviation or transcription errors during data collection.

### 2.5 Statistical Analysis

All statistical analyses were performed using SPSS version 26.0 (IBM Corp., Armonk, NY, USA) and DecisionLinnc 1.0 software (Statsape Co., Ltd, Hangzhou, Zhejiang, China). Continuous variables were summarized as mean ± SD or median (Q1–Q3), depending on their distribution assessed by the Shapiro–Wilk test and Q–Q plots. Groups were compared using the Student’s *t*-test or Mann–Whitney U test for continuous variables, and the χ^2^ or Fisher’s exact test for categorical variables. All tests were two-sided, and *p* < 0.05 was considered statistically significant. Given the exploratory nature of the study, no formal correction for multiple comparisons was applied; interpretation focused on effect sizes and 95% confidence intervals.

Missing baseline variables are summarized in **Supplementary Table 4**. Missing covariate data in the primary regression analysis were handled using multiple imputation by chained equations, with 20 imputed datasets generated. The imputation model included variables from the final multivariable model and a limited set of auxiliary baseline variables related to D-dimer levels or missingness. D-dimer was imputed on the original scale and then log-transformed [ln(x)] after imputation. No imputation was performed for the outcome variable. Pooled estimates were obtained using Rubin’s rules. As a sensitivity analysis, a complete-case logistic regression was also performed using the same final multivariable model to assess the robustness of the primary findings under an alternative missing-data handling approach (**Supplementary Tables 5,6**).

A binary logistic regression model was developed with eccentric distribution (eccentric = 1, circumferential = 0) as the dependent variable. Candidate predictors were selected based on clinical relevance and univariable results (*p *< 0.10). To reduce the risk of overfitting, model complexity was constrained to maintain an events-per-variable ratio between 8 and 10. Results are presented as odds ratios with 95% confidence intervals.

## 3. Results

### 3.1 Baseline Characteristics

A total of 156 symptomatic adult patients with a pericardial effusion were included, of whom 47 (30.1%) had eccentric (non-circumferential) and 109 (69.9%) had circumferential effusions. As summarized in Table 1, patients in the eccentric group were more likely to have a chronic disease course (>3 months: 51.06% vs. 12.84%, *p *< 0.001) and exhibited slightly higher body temperatures (median 36.6 °C vs. 36.5 °C, *p* = 0.021). The two groups were comparable in terms of sex, age, BMI, blood pressure, heart rate, and respiratory rate. Regarding comorbidities, the prevalence of pneumonia and malignancy was significantly lower in the eccentric group (pneumonia: 36.17% vs. 54.13%, *p* = 0.040; malignancy: 34.04% vs. 57.80%,* p* = 0.006). Among laboratory parameters, only D-dimer levels were significantly lower in the eccentric group (1450 [905–3200] vs. 2835 [1150–7330] ng/mL; *p* = 0.011). There were no significant differences between groups in terms of symptom presentation or incidence of cardiac tamponade.

**Table 1.** **Baseline characteristics of the study population**.

Variables		Total (n = 156)	Circumferential (n = 109)	Eccentric (n = 47)	*p*-value
Demographics					
	Male	85 (54.49%)	61 (55.96%)	24 (51.06%)	0.573
	Age (year)	62.55 ± 13.96	63.72 ± 13.04	59.83 ± 15.70	0.140
	BMI (Kg/m^2^, n = 153)	22.65 ± 3.30	22.56 ± 3.29	22.86 ± 3.33	0.608
Vital signs					
	T (℃)	36.50 (36.40–36.75)	36.50 (36.30–36.70)	36.60 (36.50–36.90)	0.021*
	Resp rate (breaths/min)	22.44 ± 5.18	22.68 ± 5.37	21.87 ± 4.69	0.866
	Heart rate (bpm)	98.97 ± 19.07	99.40 ± 16.83	97.98 ± 23.65	0.543
	SBP (mmHg)	124 (110–141.50)	124 (111–141)	123 (106–148)	0.889
	DBP (mmHg)	82.02 ± 14.73	83.03 ± 15.30	79.68 ± 13.15	0.222
Effusion duration					<0.001*
	Acute (<1 week)	47 (30.13%)	41 (37.61%)	6 (12.77%)	
	Subacute (1 week–3 months)	71 (45.51%)	54 (49.54%)	17 (36.17%)	
	Chronic (>3 months)	38 (24.36%)	14 (12.84%)	24 (51.06%)	
Previous pericardiocentesis, n (%)		29.00 (18.59%)	17.00 (15.60%)	12.00 (25.53%)	0.143
Anticoagulant use, n (%)		27.00 (17.31%)	19.00 (17.43%)	8.00 (17.02%)	0.950
Symptom, n (%)					
	Dyspnea	153 (98.08%)	108 (99.08%)	45 (95.74%)	0.216
	Chest pain	35 (22.44%)	20 (18.35%)	15 (31.91%)	0.062
	Fever	28 (17.95%)	17 (15.60%)	11 (23.40%)	0.244
	Edema	50 (32.05%)	35 (32.11%)	15 (31.91%)	0.981
	Shock	16 (10.26%)	12 (11.01%)	4 (8.51%)	0.778
	Cardiac tamponade	14 (8.97%)	10 (9.17%)	4 (8.51%)	>0.999
Comorbidity, n (%)					
	Polyserositis	116 (74.36%)	85 (77.98%)	31 (65.96%)	0.115
	Pneumonia	76 (48.72%)	59 (54.13%)	17 (36.17%)	0.040*
	Malignancy	79.00 (50.64%)	63.00 (57.80%)	16.00 (34.04%)	0.006*
	Hypoalbuminemia	49 (31.41%)	36 (33.03%)	13 (27.66%)	0.507
	Hypertension	43 (27.56%)	30 (27.52%)	13 (27.66%)	0.986
	CKD	41 (26.28%)	29 (26.61%)	12 (25.53%)	0.889
	Diabetes	33 (21.15%)	20 (18.35%)	13 (27.66%)	0.191
	Heart failure	30 (19.23%)	19 (17.43%)	11 (23.40%)	0.385
Laboratory results					
	WBC (×10^9^/L)	8.46 ± 4.64	8.85 ± 4.95	7.55 ± 3.74	0.080
	Hemoglobin (g/L)	119.75 ± 20.44	117.96 ± 18.53	123.89 ± 24.03	0.175
	Platelets (k/µL)	243.92 ± 111.76	249.20 ± 113.41	231.66 ± 108.01	0.465
	Neutrophils %	75.17 ± 10.82	75.92 ± 10.58	73.41 ± 11.27	0.139
	Albumin (g/L)	37.37 ± 5.69	37.30 ± 5.63	37.53 ± 5.90	0.682
	INR	1.11 (1.04–1.19)	1.12 (1.05–1.19)	1.09 (1.04–1.19)	0.274
	eGFR (mL/min/1.73 m^2^)	83.29 (59.26–97.48)	83.38 (58.71–96.84)	83.14 (59.99–99.28)	0.892
	PCT (ng/mL, n = 129)	0.10 (0.04–0.29)	0.11 (0.05–0.34)	0.08 (0.04–0.17)	0.194
	D-dimer (ng/mL, n = 153)	2280 (1120–5210)	2835 (1150–7330)	1450 (905–3200)	0.011*
	NT-proBNP (pg/mL, n = 135)	465 (226–1430)	428 (207–1180)	723 (333–1560)	0.181
	cTnT (µg/L, n = 145)	0.01 (0.01–0.02)	0.01 (0.01–0.02)	0.01 (0.01–0.03)	0.683

* *p* < 0.05. BMI, body mass index; SBP, systolic blood pressure; DBP, diastolic blood pressure; T, body temperature; WBC, white blood cell count; INR, international normalized ratio; eGFR, estimated glomerular filtration rate; PCT, procalcitonin; NT-proBNP, N-terminal pro–B-type natriuretic peptide; cTnT, cardiac troponin T; CKD, chronic kidney disease.

### 3.2 Logistic Regression Analysis for Factors Associated With Eccentric Distribution

As shown in Table 2, univariable logistic regression was first performed to identify potential factors associated with eccentric pericardial effusion, incorporating both variables with significant baseline differences and those considered clinically relevant. Variables with *p* < 0.10 were subsequently entered into a multivariable logistic regression model, and odds ratios (ORs) with 95% confidence intervals (CIs) were reported. In the univariable analysis, body temperature (OR 2.23, 95% CI 1.13–4.69; *p* = 0.024) and disease duration >3 months (OR 7.08, 95% CI 3.23–16.16; *p* < 0.001) were significantly associated with eccentric effusion, whereas pneumonia (OR 0.48, 95% CI 0.23–0.96; *p* = 0.041), malignancy (OR 0.38, 95% CI 0.18–0.76; *p* = 0.007), and log-transformed D-dimer levels (OR 0.65, 95% CI 0.47–0.90; *p* = 0.009) were inversely associated. In the multivariable model (adjusting for body temperature, chronic disease course, pneumonia, malignancy, and D-dimer), only body temperature (per 1 °C increase: OR 2.52, 95% CI 1.13–5.63; *p* = 0.024) and chronic course >3 months (OR 6.31, 95% CI 2.68–14.85; *p* < 0.001) remained independently associated with eccentric distribution.

**Table 2.** **Factors associated with eccentric pericardial effusion—logistic regression**.

Variable	Univariable analysis		Multivariable analysis	
	OR (95% CI)	*p*-value	OR (95% CI)	*p*-value
Gender, male	0.82 (0.41–1.63)	0.573	—	—
Age, year	0.98 (0.96–1.00)	0.112	—	—
Temperature, °C	2.23 (1.13–4.69)	0.024*	2.52 (1.13–5.63)	0.024*
Chronic duration (>3 months), yes	7.08 (3.23–16.16)	<0.001*	6.31 (2.68–14.85)	<0.001*
Previous pericardiocentesis, yes	1.86 (0.79–4.26)	0.147	—	—
Complicated with pneumonia	0.48 (0.23–0.96)	0.041*	0.49 (0.21–1.11)	0.101
Complicated with malignancy	0.38 (0.18–0.76)	0.007*	0.58 (0.25–1.33)	0.209
Complicated with hypoalbuminemia	0.78 (0.36–1.62)	0.508	—	—
ln (D‑dimer), ng/mL	0.65 (0.47–0.90)	0.009*	0.74 (0.51–1.04)	0.103
White blood cell, ×10^9^/L	0.93 (0.84–1.01)	0.114	—	—

* *p* < 0.05.

### 3.3 Echocardiographic Findings and Drainage Outcomes

As shown in Table 3, semi-quantitative echocardiographic assessment indicated that both groups had moderate-to-large effusions (>10 mm) with comparable sizes, though the site of maximal effusion depth differed significantly (*p* = 0.026). Eccentric effusions were more frequently localized to the left ventricular lateral wall (57.45% vs. 33.03%), and had a shallower anterior echo-free space (5 [3–7] vs. 11 [7–15.9] mm; *p *< 0.001). This distribution pattern made TTE-guided pericardiocentesis more challenging; consequently, while TTE guidance predominated in the circumferential group (89.9%), fluoroscopic guidance or surgical pericardial window was more frequently employed in the eccentric group (fluoroscopic 44.68% vs. 10.09%; surgical 6.38% vs. 0%; overall *p* < 0.001).

**Table 3.** **Echocardiographic findings and drainage outcomes**.

Variables			Total (n = 156)	Circumferential (n = 109)	Eccentric (n = 47)	*p*-value
Effusion data						
	Effusion size					0.809
		Moderate (10–20 mm), n (%)	62 (39.74)	44 (40.37)	18 (38.30)	
		Large (>20 mm), n (%)	94 (60.26)	65 (59.63)	29 (61.70)	
	Maximal effusion depth, mm		22 (18–26)	21 (18–25)	22.8 (19–29)	0.183
	Dominant site of maximal depth, n (%)					0.026*
		RV anterior wall	11 (7.05)	10 (9.17)	1 (2.13)	
		LV posterior wall	56 (35.90)	44 (40.37)	12 (25.53)	
		LV lateral wall	63 (40.38)	36 (33.03)	27 (57.45)	
		RV lateral wall	26 (16.67)	19 (17.43)	7 (14.89)	
	Anterior EFS (RV anterior wall), mm		9 (5–15)	11 (7–15.9)	5 (3–7)	<0.001*
Chamber diameter, mm						
	LVEDD, mm		43.64 ± 5.80	43.57 ± 5.79	43.81 ± 5.87	0.783
	LVESD, mm		29.67 ± 4.75	29.44 ± 4.56	30.21 ± 5.18	0.381
	RVD, mm		20 (18–21)	19 (18–21)	20 (18–21)	0.830
	LVEF, %		63 (59–66.5)	63 (59–67)	64 (58–66)	0.574
Sign of echo						
	Fibrin strands (echogenic), n (%)		73 (46.79)	47 (43.12)	26 (55.32)	0.161
	Septations, n (%)		22 (14.10)	11 (10.09)	11 (23.40)	0.028*
	Pericardial thickening, n (%)		20 (12.82)	10 (9.17)	10 (21.28)	0.038*
	Swinging heart, n (%)		55 (35.26)	44 (40.37)	11 (23.40)	0.042*
	RV diastolic collapse, n (%)		49 (31.41)	43 (39.45)	6 (12.77)	<0.001*
	Constrictive pericarditis, n (%)		5 (3.21)	1 (0.92)	4 (8.51)	0.029*
Drainage approach						<0.001*
	TTE-guided, n (%)		121 (77.56)	98 (89.91)	23 (48.94)	
	Fluoroscopy-guided, n (%)		32 (20.51)	11 (10.09)	21 (44.68)	
	Surgical pericardial window, n (%)		3 (1.92)	0 (0.00)	3 (6.38)	
Major complication						
	Bleeding, n (%)		2 (1.28)	1 (0.92)	1 (2.13)	0.513
Drainage outcomes						
	Drainage duration, days		3 (2–5)	3 (2–5)	4 (2–5)	0.576
	Drainage volume, mL		838.5 (555–1235)	914 (600–1260)	600 (360–1150)	0.005*
	Poor drainage, n (%)		30 (19.23)	9 (8.26)	21 (44.68)	<0.001*

* *p* < 0.05. LV, left ventricle; RV, right ventricle; TTE, transthoracic echocardiography; EFS, echo-free space; LVEDD, left ventricular end-diastolic diameter; LVESD, left ventricular end-systolic diameter; RVD, right ventricular diameter; LVEF, left ventricular ejection fraction; “Swinging heart” refers to a visually appreciable to-and-fro motion of the heart, typically the left ventricle, within a large pericardial effusion as observed on TTE.

Echocardiographic features such as septations (23.40% vs. 10.09%, *p* = 0.028), pericardial thickening (21.28% vs. 9.17%, *p* = 0.038), and constrictive pericarditis (8.51% vs. 0.92%, *p* = 0.029) were more common in eccentric cases. In exploratory extended models that additionally included septations and/or pericardial thickening, all VIFs were <5, and the main multivariable findings were unchanged, with body temperature and chronic duration >3 months remaining the only independent associated factors (**Supplementary Table 7**). In contrast, classic tamponade signs were less frequent, including RV diastolic collapse (12.77% vs. 39.45%, *p* < 0.001) and swinging heart motion (23.40% vs. 40.37%, *p* = 0.042). Cardiac chamber dimensions and LVEF were comparable between groups. Although catheter dwell time did not differ significantly, the total drainage volume was smaller in the eccentric group (600 [360–1150] vs. 914 [600–1260] mL; *p* = 0.005), and the incidence of poor drainage was markedly higher (44.68% vs. 8.26%, *p* < 0.001). Major hemorrhagic complications were rare and similar between groups (2.13% vs. 0.92%, *p* = 0.513), and no procedure-related deaths occurred.

### 3.4 Pericardial Fluid Analysis and Diagnostic Yield

As summarized in Table 4, the macroscopic appearance of pericardial fluid differed significantly between groups (*p* < 0.001): the eccentric group more frequently exhibited yellow and less often bloody effusions. The eccentric group also showed lower cell counts (total, nucleated, red blood cells, and polymorphonuclear cells; *p *≤ 0.019) and lower biochemical activity, with reduced LDH, ADA, bilirubin, and effusion-to-serum LDH ratios (all *p *≤ 0.009). The Rivalta test and cytological positivity for malignancy were both lower in the eccentric group (*p* = 0.029 and *p* = 0.019, respectively). The overall diagnostic yield of pericardial fluid cytology or microbiology was numerically lower in the eccentric group (29.79% vs. 44.95%), though the difference did not reach statistical significance (*p* = 0.076). Etiology-stratified and malignancy-adjusted supplementary analyses are presented in **Supplementary Tables 8,9**. In these analyses, the differences in cytology positivity and fluid-based diagnostic yield were largely explained by the differing prevalence of malignant effusions between groups.

**Table 4.** **Pericardial fluid analysis and diagnostic yield**.

Variable		Total (n = 156)	Circumferential (n = 109)	Eccentric (n = 47)	*p*-value
Color					<0.001*
	Red, n (%)	90 (57.69)	73 (66.97)	17 (36.17)	
	Yellow, n (%)	62 (39.74)	35 (32.11)	27 (57.45)	
	Greenish, n (%)	3 (1.92)	1 (0.92)	2 (4.26)	
	White, n (%)	1 (0.64)	0 (0.00)	1 (2.13)	
Clarity					0.067
	Clear, n (%)	146 (93.59)	105 (96.33)	41 (87.23)	
	Turbid, n (%)	10 (6.41)	4 (3.67)	6 (12.77)	
Cell numbers					
	Total cell count, ×10^9^/L	90.36 (5.67–1313.25)	380.35 (8.10–1471.70)	11.26 (1.59–317.16)	<0.001*
	Red blood cells, ×10^9^/L	87.00 (3.00–1270.50)	375.00 (6.58–1471.00)	10.03 (1.00–315.00)	0.001*
	Total nucleated cells, ×10^6^/L	2097 (684.50–4725)	2378 (1169–4541)	904 (155–5219)	0.019*
	Polymorphonucleated cells, ×10^6^/L	395.5 (61.5–1701.0)	529.0 (145.0–1832.0)	79.0 (20.0–1057.0)	0.008*
	Mononucleated cells, ×10^6^/L	1148.5 (409.5–2595.5)	1376.0 (574.0–2485.0)	786.0 (110.0–3033.0)	0.088
Biochemical measurand					
	Total protein, g/L	51 (45–56)	52 (47–56)	49 (41–57)	0.320
	Albumin, g/L	30.90 ± 7.05	31.03 ± 6.10	30.62 ± 8.95	0.775
	Globulin, g/L	19 (16–23)	20 (17–23)	18 (13–21)	0.015*
	Bilirubin, μmol/L	20.3 (10.1–57.0)	29.4 (11.5–67.4)	12.2 (8.0–26.8)	<0.001*
	LDH, U/L	667.5 (259.5–1654.5)	786 (407–1732)	324 (167–937)	<0.001*
	ADA, U/L	13.45 (7.30–28.90)	15.90 (8.20–30.20)	9.60 (5.10–15.50)	0.009*
	LDH ratio (PF/serum)	2.60 (1.13–5.07)	2.92 (1.48–5.24)	1.22 (0.77–3.72)	0.003*
	Protein ratio (PF/serum)	0.77 (0.68–0.86)	0.78 (0.69–0.86)	0.74 (0.64–0.85)	0.076
	Light’s criteria positive, n (%)	153 (98.08)	108 (99.08)	45 (95.74)	0.216
	Rivalta positive, n (%)	151 (96.79)	108 (99.08)	43 (91.49)	0.029*
Microbiology positive, n (%)		5 (3.21)	2 (1.83)	3 (6.38)	0.161
	Mycobacterium tuberculosis, n (%)	2 (1.28)	1 (0.92)	1 (2.13)	
	Other bacteria, n (%)	3 (1.92)	1 (0.92)	2 (4.26)	
Cytology positive, n (%)		58 (37.18)	47 (43.12)	11 (23.40)	0.019*
Diagnostic yield (fluid-based), n/N (%)		63 (40.38)	49 (44.95)	14 (29.79)	0.076

* *p* < 0.05. LDH, lactate dehydrogenase; ADA, adenosine deaminase; PF, pericardial fluid; Light’s criteria positive: PF/serum protein ≥0.5, PF/serum LDH ≥0.6, or PF LDH ≥2/3 the upper limit of normal; Diagnostic yield was defined as the proportion of cases with positive microbiological or cytological results among all specimens submitted for laboratory testing.

### 3.5 Etiology and Outcomes

**Fig. 2.**
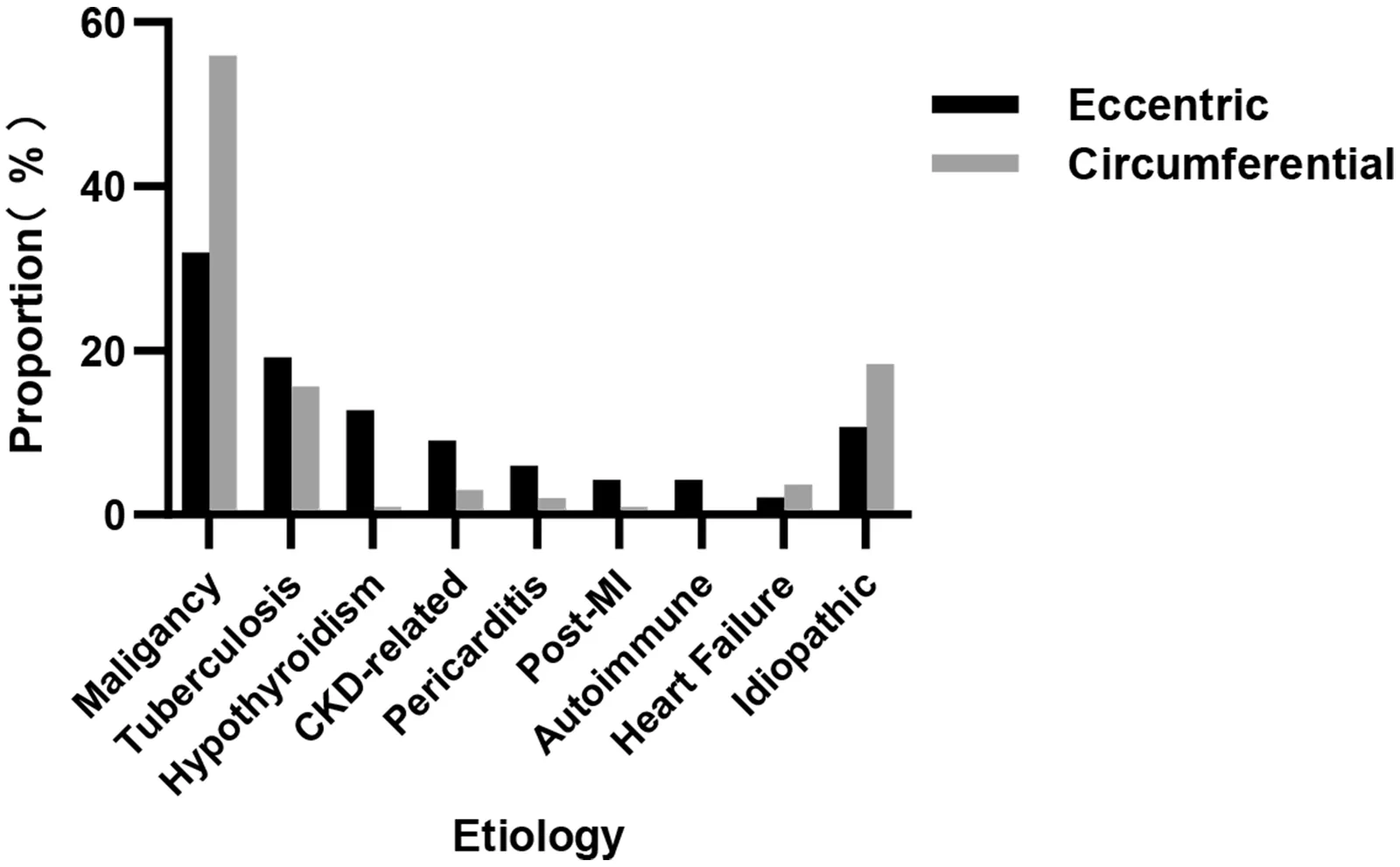
**Etiologic distribution by group (eccentric vs circumferential)**. CKD-related, chronic kidney disease related; Post-MI, post-myocardial infarction syndrome.

As shown in Table 5, the etiologic distribution of pericardial effusion differed significantly between groups (*p* < 0.001). While malignancy remained the leading cause in both groups, it was more frequent in the circumferential group (55.96% vs. 31.91%). In contrast, the eccentric group demonstrated a more heterogeneous etiologic spectrum (see Fig. 2), with higher proportions of hypothyroidism-related (12.77% vs. 0.92%) and CKD-related (8.51% vs. 2.75%) effusions. In-hospital outcomes, including length of stay, tamponade occurrence, cardiac mortality, and all-cause mortality, did not differ significantly between groups (all *p *> 0.05). The incidence of cardiac tamponade was comparable (8.51% vs. 9.17%, *p *> 0.999), as were cardiac death (4.26% vs. 3.67%) and all-cause death (6.38% vs. 5.50%).

**Table 5.** **Etiologic distribution and clinical outcomes**.

Variable			Total (n = 156)	Circumferential (n = 109)	Eccentric (n = 47)	*p*-value
Etiology						<0.001*
	Malignancy, n (%)		76 (48.72)	61 (55.96)	15 (31.91)	
		Lung cancer, n/N	61/76	51/61	10/15	
		Breast cancer, n/N	6/76	4/61	2/15	
		Lymphoma, n/N	3/76	1/61	2/15	
		Others, n/N	6/76	5/61	1/15	
	Tuberculosis, n (%)		26 (16.67)	17 (15.60)	9 (19.15)	
	CKD-related, n (%)		7 (4.49)	3 (2.75)	4 (8.51)	
	Hypothyroidism, n (%)		7 (4.49)	1 (0.92)	6 (12.77)	
	Pericarditis, n (%)		5 (3.21)	2 (1.83)	3 (6.38)	
	Heart failure, n (%)		5 (3.21)	4 (3.67)	1 (2.13)	
	Post-MI syndrome, n (%)		3 (1.92)	1 (0.92)	2 (4.26)	
	Autoimmune, n (%)		2 (1.28)	0 (0.00)	2 (4.26)	
	Idiopathic, n (%)		25 (16.03)	20 (18.35)	5 (10.64)	
In-hospital outcomes						
	Length of stay, days		8 (6–13)	8 (5.50–13)	9 (7–13)	0.314
	Cardiac tamponade, n (%)		14 (8.97)	10 (9.17)	4 (8.51)	>0.999
	Cardiac death, n (%)		6 (3.85)	4 (3.67)	2 (4.26)	>0.999
	All-cause death, n (%)		9 (5.77)	6 (5.50)	3 (6.38)	>0.999
90-day follow-up outcomes						
	Effusion recurrence, n/N (%)		58/147	36/103 (34.95)	22/44 (50.00)	0.127
	Readmission, n/N (%)		44/147	28/103 (27.18)	16/44 (36.36)	0.360
	Repeat puncture, n/N (%)		18/147	13/103 (12.62)	5/44 (11.36)	>0.999
	90-day Death, n/N (%)		10/147	6/103 (5.83)	4/44 (9.09)	0.487

* *p* < 0.05. CKD-related, chronic kidney disease related; Post-MI syndrome, post-myocardial infarction syndrome.

At 90-day follow-up, both groups exhibited a relatively high rate of effusion recurrence (eccentric 50.00% [22/44] vs. circumferential 34.95% [36/103]), though the difference did not reach statistical significance (*p *= 0.127). Rates of rehospitalization and repeat pericardiocentesis were also similar. Exploratory subgroup analyses of 90-day recurrence by etiology and drainage strategy are presented in **Supplementary Tables 10–12**. These analyses suggested that the numerically higher recurrence rate in eccentric effusions was most evident in malignant cases, whereas no clear heterogeneity by drainage strategy was observed. In an exploratory 1:1 propensity score–matched analysis among discharged patients, baseline balance improved overall, although a modest residual imbalance remained for age and malignancy. In the matched cohort, the eccentric group continued to show numerically higher recurrence, readmission, and 90-day mortality rates, but none of these differences reached statistical significance (**Supplementary Tables 13,14**).

## 4. Discussion

To our knowledge, this is the first study to systematically compare eccentric and circumferential pericardial effusions in a symptomatic drainage cohort, highlighting their distinct clinical, imaging, and procedural characteristics.

In our cohort, eccentric pericardial effusion accounted for 30.1% (47/156) of all drainage cases, which is slightly lower than the 41.2% (40/97) reported by Kim et al. [11] for “Circumferential uneven” and loculated effusions patterns among evaluable drainage procedures. We found that a chronic course and elevated body temperature were independently associated with eccentric pericardial effusion in multivariable logistic regression analysis. Structural pericardial changes such as septations (23.40% vs. 10.09%, *p* = 0.028), pericardial thickening (21.28% vs. 9.17%, *p* = 0.038), and constrictive pericarditis (8.51% vs. 0.92%, *p* = 0.029) were more frequently observed in the eccentric group. A plausible explanation is that elevated body temperature may reflect active pericardial inflammation, and chronic or recurrent inflammation facilitates fibrinous exudation, adhesion formation, and partial septation, ultimately resulting in localized fluid collections and eccentric distribution. This inflammation-fibrous-compartmentalization pathway has been postulated in previous reviews and guidelines [1,4,20], and our study provides the first quantitative data that are consistent with this proposed mechanism. However, these associations should be interpreted cautiously. Chronic disease course is conceptually related to the presumed pathophysiology of eccentric effusions, and reverse causality cannot be excluded in this retrospective study. Likewise, although body temperature remained statistically significant in multivariable analysis, the absolute between-group difference was small, and its biological relevance should not be overinterpreted. Overall, our findings are consistent with, but do not prove, the hypothesized inflammation-fibrosis-compartmentalization pathway in eccentric pericardial effusions.

The LV lateral wall was identified as the predominant site of fluid accumulation in eccentric cases (57.45% vs. 33.03%). In contrast, Kim et al. [11] reported that 67.5% (27/40) of circumferentially uneven effusions were predominantly posterior. This discrepancy likely arises from differences in classification, as their study simply categorized nonuniform effusions as “mainly posterior”, “mainly RA side”, or “mainly RV side”, without differentiating between posterior (LV posterior wall) and lateral wall localization as in our study. We also observed a lower frequency of right ventricular diastolic collapse in eccentric cases compared with circumferential ones, likely because eccentric effusions exert focal pressure predominantly on the LV lateral wall, reducing the sensitivity of classic tamponade signs such as right atrial or right ventricular diastolic collapse. This finding aligns with prior reports describing postoperative loculated (non-circumferential) effusions [21,22,23].

Our findings underscore the importance of adapting the pericardiocentesis approach to the anatomical distribution of the effusion. Consistent with current guidelines recommending TTE guidance as the first-line technique [1], the majority of circumferential effusions in our study were successfully drained under TTE guidance. However, a key observation was that nearly half of eccentric effusions required fluoroscopic guidance (44.68% vs. 10.09%, *p* < 0.001). This was primarily due to the shallow anterior fluid layer and the frequent presence of fibrous septations, which limited the availability of a safe acoustic window for conventional TTE-guided puncture. In such situations, fluoroscopic guidance offers significant procedural advantages. As demonstrated both in our practice and previous studies, fluoroscopy enables real-time trajectory planning and safe catheter placement [13,15,16]. The needle and guidewire can be continuously visualized in relation to cardiac contours, and small-volume contrast injection facilitates confirmation of entry into the intended pericardial space. This approach effectively reduces the risk of inadvertent injury to the myocardium, coronary arteries, or adjacent organs. Molkara et al. [10] described combined echo/fluoroscopic techniques using nonstandard entry sites to reach noncircumferential effusions, highlighting creative routes for difficult cases. Similarly, interventional series have shown that when an effusion is located posteriorly, a transhepatic (inferior) puncture approach under fluoroscopy can effectively reach the fluid, which would be nearly impossible via the usual subxiphoid or apical echo-guided routes [11]. Our findings reinforce these observations: when echocardiography suggests that a pericardial effusion is predominantly localized to the posterior or lateral wall and no safe acoustic window is available, fluoroscopic guidance should be considered. At the same time, the procedural differences observed in this study should be cautiously interpreted. Variables such as guidance modality and drainage performance are, to some extent, inherently influenced by effusion geometry. Accordingly, these findings are better understood as real-world procedural correlates and practical consequences of eccentric morphology in an invasive-management setting, rather than as wholly independent, unexpected, or causal discoveries. Their primary value is in improving pre-procedural planning and aiding the interpretation of drainage performance in patients with complex effusion anatomy.

Identifying the underlying etiology is a key objective in the management of pericardial effusion, and our study provides potential diagnostic insights for clinical practice. Although malignancy remains the most common overall single cause of pericardial effusion, the overall etiologic distribution differed significantly between eccentric and circumferential effusions (overall *p* < 0.001). Compared with circumferential effusions, eccentric effusions showed a lower proportion of malignant etiologies (31.91% vs. 55.96%). As illustrated in Fig. 2, the etiological spectrum of eccentric effusions was more heterogeneous, with a relatively higher proportion of non-malignant chronic conditions such as hypothyroidism, chronic kidney disease, and autoimmune disorders. This etiological divergence was also reflected in the biochemical profiles of the pericardial fluid. We also observed that cytology positivity and overall fluid-based diagnostic yield were lower in eccentric than in circumferential effusions. However, these findings should be interpreted cautiously, because they may partly reflect the differing etiologic composition between the two groups, particularly the lower prevalence of malignant effusions in the eccentric group. In our supplementary analyses, after etiology stratification and adjustment for malignancy, the differences in cytology positivity and fluid-based diagnostic yield were markedly attenuated, suggesting that these overall differences were largely driven by the imbalance in malignancy rather than by effusion morphology alone.

From a diagnostic standpoint, this observation has clinical importance. For circumferential effusions of unclear origin, clinicians should maintain a high index of suspicion for underlying malignancy. Cytological examination in our cohort demonstrated a robust diagnostic yield, with a positivity rate of 43.12% in the circumferential group and an overall positivity of 37.18%. Previous studies have similarly underscored the pivotal role of cytologic analysis in identifying malignant pericardial effusions, although reported diagnostic yields have ranged from 15% to 24.2%, which is lower than our findings [24,25,26]. The higher diagnostic yield in our study may be partly explained by the fact that all enrolled patients had moderate-to-large pericardial effusions, which are often indicative of malignant etiologies [27]. In contrast, eccentric pericardial effusions in our cohort were more commonly associated with non-malignant chronic diseases. For these patients, a comprehensive evaluation of metabolic and autoimmune factors is essential, including assessment of thyroid function, renal function, and autoantibody screening.

Finally, although in-hospital and short-term outcomes did not differ significantly between the two groups, both demonstrated a substantial recurrence rate of pericardial effusions within 90 days (eccentric 50.00% vs. circumferential 34.95%; *p* = 0.127). This finding is consistent with prior reports documenting recurrence rates of 30%–61.5% following pericardial drainage [28,29,30], underscoring the need for vigilant follow-up regardless of the morphology of the effusion. However, given the exploratory nature of the study and the modest size of the eccentric subgroup, this outcome analysis was likely underpowered, and the absence of statistical significance should not be interpreted as evidence of the absence of differences in prognosis.

### Limitations

This study has several limitations. First, it was a single-center retrospective analysis restricted to symptomatic patients undergoing invasive evaluation or treatment; therefore, selection bias is unavoidable, and the findings are not generalizable to small, asymptomatic, or conservatively managed pericardial effusions. Second, because no established reference definition for eccentric effusion is available, the echocardiographic classification used in this study should be regarded as an operational, study-specific framework rather than a formally validated standard. Formal interobserver and intraobserver reproducibility analyses were not incorporated into the original retrospective workflow, so classification bias cannot be fully excluded. Third, given the exploratory nature of the study and the modest size of the eccentric subgroup, residual confounding and small-sample bias remain possible despite multivariable adjustment. Fourth, drainage strategies were not fully standardized, and operator-related factors were not systematically recorded; therefore, their potential influence on drainage performance could not be fully assessed. In addition, the classification of pericardial effusion by disease course used in this study was based on an earlier guideline framework available at the time of study design. The 2025 ESC Guidelines use different temporal categories and advise against directly applying pleural-fluid Light’s criteria to pericardial effusions; these differences should be considered when interpreting our findings. Finally, some etiologic categories were based partly on clinical adjudication, and etiologic misclassification cannot be fully excluded. Future multicenter prospective studies with predefined imaging criteria, formal reproducibility assessment, and larger cohorts are warranted to validate these findings.

## 5. Conclusions

In symptomatic patients undergoing invasive management for pericardial effusion, eccentric morphology was independently associated with a chronic disease course and higher body temperature. Compared with circumferential effusions, eccentric effusions more often demonstrated left ventricular lateral predominance, fewer classic echocardiographic signs of tamponade, and greater procedural reliance on fluoroscopic guidance, with a higher likelihood of poor drainage. In addition, their etiologic spectrum was more heterogeneous and less often malignant. These findings may help improve preprocedural planning and guide etiologic evaluation in patients with complex pericardial effusion anatomy.

## Data Availability

The datasets used and analyzed during the current study are available upon reasonable request, subject to approval by both the corresponding author and the institutional ethics committee.

## Abbreviations

PEff, pericardial effusion; TTE, transthoracic echocardiography; EFS, echo-free space; LV, left ventricle; RV, right ventricle; LVEDD, left ventricular end-diastolic diameter; LVESD, left ventricular end-systolic diameter; RVD, right ventricular diameter; CKD, chronic kidney disease.

## Supplementary Material

The Supplementary Material includes **Supplementary Table 1** providing the STROBE checklist; **Supplementary Tables 2,3** evaluating the distribution of dominant-to-contralateral echo-free-space ratios and the sensitivity of the operational 2:1 cutoff used to define eccentric pericardial effusion; **Supplementary Table 4** summarizing missing variables in the full cohort; **Supplementary Tables 5,6** presenting complete-case and multiple-imputation sensitivity analyses for the logistic regression model; **Supplementary Table 7** showing exploratory multivariable models additionally incorporating echocardiographic morphologic variables; **Supplementary Tables 8,9** presenting etiology-stratified and malignancy-adjusted analyses of key pericardial fluid diagnostic findings; **Supplementary Tables 10–12** reporting exploratory subgroup and interaction analyses of 90-day effusion recurrence according to etiology and drainage strategy; and **Supplementary Tables 13,14** summarizing baseline characteristics and 90-day outcomes before and after 1:1 propensity score matching among patients discharged alive. Supplementary material associated with this article can be found, in the online version, at https://doi.org/10.31083/RCM48796.
