# Supplementary material for: Clinical, Echocardiographic, and Procedural Characteristics of Eccentric Versus Circumferential Pericardial Effusions in a Symptomatic Drainage Cohort
Source: Rev Cardiovasc Med. 2026 Aug 19;27(8):48796. doi: 10.31083/RCM48796 (PMC13540029; doi:10.31083/RCM48796)
Supplement: Supplementary file 1 [file 2153-8174-27-8-48796-s1.zip › Supplementary Material.docx]

**Supplementary Table 1. STROBE Statement—Checklist of items that should be included in reports of cohort studies**

|  | Item No | Recommendation | | Page  No |
| --- | --- | --- | --- | --- |
| **Title and abstract** | 1 | (*a*) Indicate the study’s design with a commonly used term in the title or the abstract | | 1 |
|  |  | (*b*) Provide in the abstract an informative and balanced summary of what was done and what was found | | 1 |
| Introduction | | | |  |
| Background/rationale | 2 | Explain the scientific background and rationale for the investigation being reported | | 1–2 |
| Objectives | 3 | State specific objectives, including any prespecified hypotheses | | 2 |
| Methods | | | |  |
| Study design | 4 | Present key elements of study design early in the paper | | 2 |
| Setting | 5 | Describe the setting, locations, and relevant dates, including periods of recruitment, exposure, follow-up, and data collection | | 2, 4 |
| Participants | 6 | (*a*) Give the eligibility criteria, and the sources and methods of selection of participants. Describe methods of follow-up | | 2, 4 |
|  |  | (*b*) For matched studies, give matching criteria and number of exposed and unexposed | | N/A |
| Variables | 7 | Clearly define all outcomes, exposures, predictors, potential confounders, and effect modifiers. Give diagnostic criteria, if applicable | | 2–4 |
| Data sources/ measurement | 8* | For each variable of interest, give sources of data and details of methods of assessment (measurement). Describe comparability of assessment methods if there is more than one group | | 2–4 |
| Bias | 9 | Describe any efforts to address potential sources of bias | | 4, 11 |
| Study size | 10 | Explain how the study size was arrived at | | 2 |
| Quantitative variables | 11 | Explain how quantitative variables were handled in the analyses. If applicable, describe which groupings were chosen and why | | 4–5 |
| Statistical methods | 12 | (*a*) Describe all statistical methods, including those used to control for confounding | | 4–5 |
|  |  | (*b*) Describe any methods used to examine subgroups and interactions | | 8–9 |
|  |  | (*c*) Explain how missing data were addressed | | 4–5 |
|  |  | (*d*) If applicable, explain how loss to follow-up was addressed | | 4, 8–9 |
|  |  | (*e*) Describe any sensitivity analyses | | 3, 5, 8–9 |
| Results | | | |  |
| Participants | 13* | (a) Report numbers of individuals at each stage of study—eg numbers potentially eligible, examined for eligibility, confirmed eligible, included in the study, completing follow-up, and analysed | | 2–3, 8–9 |
|  |  | (b) Give reasons for non-participation at each stage | | 2 |
|  |  | (c) Consider use of a flow diagram | | 3 |
| Descriptive data | 14* | (a) Give characteristics of study participants (eg demographic, clinical, social) and information on exposures and potential confounders | | 5–6 |
|  |  | (b) Indicate number of participants with missing data for each variable of interest | Supplementary Table 4 | |
|  |  | (c) Summarise follow-up time (eg, average and total amount) | | 4, 8–9 |
| Outcome data | 15* | Report numbers of outcome events or summary measures over time | | 8–9 |
| Main results | 16 | (*a*) Give unadjusted estimates and, if applicable, confounder-adjusted estimates and their precision (eg, 95% confidence interval). Make clear which confounders were adjusted for and why they were included | | 5, 7 |
|  |  | (*b*) Report category boundaries when continuous variables were categorized | | 4, 6–7 |
|  |  | (*c*) If relevant, consider translating estimates of relative risk into absolute risk for a meaningful time period | | N/A |
| Other analyses | 17 | Report other analyses done—eg analyses of subgroups and interactions, and sensitivity analyses | | 5, 8–9 |
| Discussion | | | |  |
| Key results | 18 | Summarise key results with reference to study objectives | | 9–11 |
| Limitations | 19 | Discuss limitations of the study, taking into account sources of potential bias or imprecision. Discuss both direction and magnitude of any potential bias | | 11 |
| Interpretation | 20 | Give a cautious overall interpretation of results considering objectives, limitations, multiplicity of analyses, results from similar studies, and other relevant evidence | | 9–11 |
| Generalisability | 21 | Discuss the generalisability (external validity) of the study results | | 11 |
| Other information | | | |  |
| Funding | 22 | Give the source of funding and the role of the funders for the present study and, if applicable, for the original study on which the present article is based | | 12 |

*Give information separately for exposed and unexposed groups.

**Note:** An Explanation and Elaboration article discusses each checklist item and gives methodological background and published examples of transparent reporting. The STROBE checklist is best used in conjunction with this article (freely available on the Web sites of PLoS Medicine at http://www.plosmedicine.org/, Annals of Internal Medicine at http://www.annals.org/, and Epidemiology at http://www.epidem.com/). Information on the STROBE Initiative is available at http://www.strobe-statement.org.

Supplementary Table 2. Distribution of cases according to dominant-to-contralateral EFS ratio

| **Ratio range** | **Non-borderline**  **<1.8** | **Borderline**  **1.8–2.2** | **Non-borderline**  **>2.2** |
| --- | --- | --- | --- |
| Eccentric, n (%) | 0 | 2 | 45 |
| Circumferential, n (%) | 95 | 14 | 0 |
| All cases, n (%) | 95 | 16 | 45 |

**Supplementary Table 3. Sensitivity analysis for the operational 2:1 cutoff used to define eccentric pericardial effusion**

| **Analysis cohort** | **Cutoff rule** | **Excluded borderline (cases, n)** | **Final**  **(n)** | **Eccentric**  **(n)** | **multivariable logistic regression** | | | |
| --- | --- | --- | --- | --- | --- | --- | --- | --- |
|  |  |  |  |  | **Variable** | **OR (95% CI)** | ***p*-value** |  |
| Original analytic cohort | Dominant/contralateral EFS  ratio ≥2.0 | 0 | 156 | 47 | Temperature, °C | 2.52 (1.13-5.63) | 0.024* | Reference model |
|  |  |  |  |  | Chronic duration (>3 months), yes | 6.31 (2.68-14.85) | <0.001* |  |
|  |  |  |  |  | Complicated with pneumonia, yes | 0.49 (0.21-1.11) | 0.101 |  |
|  |  |  |  |  | Complicated with malignancy, yes | 0.58 (0.25-1.33) | 0.209 |  |
|  |  |  |  |  | ln (D-dimer), ng/mL | 0.74 (0.51-1.04) | 0.103 |  |
| High-confidence cohort | Excluding borderline cases with ratio 1.8–2.2 | 16 | 140 | 45 | Temperature, °C | 2.99 (1.23-7.32) | 0.016* | Materially unchanged |
|  |  |  |  |  | Chronic duration (>3 months), yes | 7.35 (2.94-18.35) | <0.001* |  |
|  |  |  |  |  | Complicated with pneumonia, yes | 0.44 (0.18-1.09) | 0.075 |  |
|  |  |  |  |  | Complicated with malignancy, yes | 0.56 (0.24-1.34) | 0.192 |  |
|  |  |  |  |  | ln (D-dimer), ng/mL | 0.68 (0.46-1.02) | 0.063 |  |

Supplementary Table 4. Missing variables in the full cohort

| Missing variables | Miss.freq | Miss.percentage(%) |
| --- | --- | --- |
| BMI | 3 | 1.92 |
| D-dimer | 3 | 1.92 |
| cTNT | 11 | 7.05 |
| NT-proBNP | 21 | 13.46 |
| PCT | 27 | 17.31 |
| Drainage time (day) | 1 | 0.64 |
| Drainage volume | 1 | 0.64 |

BMI, body mass index; cTNT, cardiac troponin T; NT-proBNP, N-terminal pro–B-type natriuretic peptide; PCT, procalcitonin.

**Supplementary Table 5. Sensitivity analysis using complete cases for variables included in the logistic regression model**

|  | Univariable Analysis  (n=153) | | Multivariable Analysis  (n=153) | | |
| --- | --- | --- | --- | --- | --- |
| Variable | OR (95% CI) | *p*-value | OR (95% CI) | *p*-value | |
| Gender, male | 0.83 (0.42-1.66) | 0.599 | — | | — |
| Age, year | 0.98 (0.96-1.01) | 0.126 | — | | — |
| Temperature, °C | 2.14 (1.08-4.50) | 0.032* | 2.52 (1.16-5.92) | | 0.024* |
| Chronic duration (>3 months), yes | 6.85 (3.12-15.66) | <0.001* | 6.31 (2.72-15.25) | | <0.001* |
| Previous pericardiocentesis, yes | 1.80 (0.77-4.13) | 0.170 | — | | — |
| Complicated with pneumonia | 0.51 (0.25-1.02) | 0.059 | 0.50 (0.21-1.13) | | 0.101 |
| Complicated with malignancy | 0.40 (0.19-0.80) | 0.011* | 0.59 (0.26-1.34) | | 0.210 |
| Complicated with hypoalbuminemia | 0.81 (0.37-1.70) | 0.585 | — | | — |
| ln (D-dimer), ng/mL | 0.65 (0.47-0.89) | 0.009* | 0.74 (0.50-1.05) | | 0.103 |
| White blood cell, ×10⁹/L | 0.94 (0.85-1.02) | 0.162 | — | | — |

Supplementary Table 6. Sensitivity analysis of multivariable logistic regression for factors associated with eccentric pericardial effusion

| Variable | Primary analysis: multiple imputation (n=156) OR (95% CI) | *p*-value | Sensitivity analysis: complete-case analysis (n=153) OR (95% CI) | *p*-value |
| --- | --- | --- | --- | --- |
| Temperature, °C | 2.52 (1.13-5.63) | 0.024* | 2.52 (1.16-5.92) | 0.024* |
| Chronic duration (>3 months), yes | 6.31 (2.68-14.85) | <0.001* | 6.31 (2.72-15.25) | <0.001* |
| Complicated with pneumonia | 0.49 (0.21-1.11) | 0.101 | 0.50 (0.21-1.13) | 0.101 |
| Complicated with malignancy | 0.58 (0.25-1.33) | 0.209 | 0.59 (0.26-1.34) | 0.210 |
| ln (D-dimer), ng/mL | 0.74(0.51-1.04) | 0.103 | 0.74 (0.50-1.05) | 0.103 |

**Supplementary Table 7. Exploratory multivariable models including echocardiographic morphologic variables**

| Variable | Primary model  OR (95% CI) | *p-*value | Extended model  OR (95% CI) | *p-*value | VIF |
| --- | --- | --- | --- | --- | --- |
| Temperature, °C | 2.52 (1.13-5.63) | 0.024* | 2.44 (1.12-5.78) | 0.031* | 1.059 |
| Chronic duration (>3 months), yes | 6.31 (2.68-14.85) | <0.001* | 5.74 (2.40-14.33) | <0.001* | 1.140 |
| Complicated with pneumonia, yes | 0.49 (0.21-1.11) | 0.101 | 0.47 (0.20-1.08) | 0.092 | 1.050 |
| Complicated with malignancy, yes | 0.58 (0.25-1.33) | 0.209 | 0.61 (0.26-1.39) | 0.256 | 1.118 |
| ln (D-dimer), ng/mL | 0.74 (0.51-1.04) | 0.103 | 0.72 (0.48-1.03) | 0.083 | 1.106 |
| Septations, yes | — | — | 1.62 (0.45-5.70) | 0.448 | 1.495 |
| Pericardial thickening, yes | — | — | 1.17 (0.29-4.58) | 0.814 | 1.553 |

Exploratory models were fitted to assess whether addition of echocardiographic morphologic variables altered the main multivariable findings. Variance inflation factors for all included predictors were <5.

**Supplementary Table 8. Etiology-stratified comparisons of key pericardial fluid parameters by effusion morphology**

| Variable | Malignant | |  | Non-malignant | |  |
| --- | --- | --- | --- | --- | --- | --- |
|  | Circumferential  (n=61) | Eccentric  (n=15) | p-value | Circumferential  (n=48) | Eccentric  (n=32) | p-value |
| LDH, median (IQR) | 898.0 (1593.0) | 441.0 (727.0) | 0.044* | 729.0 (1344.5) | 294.5 (771.0) | 0.021* |
| ADA, median (IQR) | 12.90 (16.50) | 6.00 (9.90) | 0.037* | 23.10 (30.15) | 9.65 (22.25) | 0.019* |
| Total cells, ×10^6^/L, median (IQR), | 615863.0 (1827695.0) | 55943.0 (888665.0) | 0.163 | 180662.0 (1016744.5) | 6108.0 (69843.5) | 0.002* |
| Cytology positive, n/N (%) | 77.05% (47 / 61) | 73.33% (11 / 15) | 0.762 | 0% (0 / 48) | 0% (0 / 32) | - |
| Fluid-based diagnostic yield, n/N (%) | 77.05% (47 / 61) | 73.33% (11 / 15) | 0.762 | 4.17% (2 / 48) | 9.38% (3 / 32) | 0.384 |

**Supplementary Table 9. Malignancy-adjusted analyses of key pericardial fluid diagnostic outcomes**

| Outcome | Unadjusted OR for eccentric morphology  OR (95% CI) | p value | Adjusted OR for eccentric morphology*  OR (95% CI) | p value |
| --- | --- | --- | --- | --- |
| Cytology positive | 0.40 (0.18-0.85) | 0.022* | Not estimable due to data separation† | - |
| Fluid-based diagnostic yield | 0.52 (0.24-1.06) | 0.079 | 1.19 (0.41-3.75) | 0.761 |

*The adjusted model included effusion morphology and malignancy status (malignant vs non-malignant etiology).

†The adjusted OR for cytology positivity could not be reliably estimated because of data separation, as no cytology-positive cases occurred in the non-malignant subgroup.

Supplementary Table 10. Exploratory subgroup analyses of 90-day effusion recurrence by etiology and drainage strategy

| Subgroup | Circumferential recurrence, n/N (%) | Eccentric  recurrence, n/N (%) | *p*-value |
| --- | --- | --- | --- |
| By etiology |  |  |  |
| Malignant | 21/57 (36.84%) | 11/14 (78.57%) | 0.005* |
| Non-malignant | 15/46 (32.61%) | 11/30 (36.67%) | 0.715 |
|  |  |  |  |
| By drainage strategy |  |  |  |
| TTE-guided | 31/92 (33.70%) | 10/20 (50%) | 0.170 |
| Fluoroscopy-guided/Surgical  pericardial window, | 5/11 (45.45%) | 12/24 (50.00%) | 0.803 |

**Supplementary Table 11. Interaction model for etiology**

| Variable in interaction model* | OR (95% CI) | *p-*value |
| --- | --- | --- |
| Eccentric morphology | 1.19 (0.45-3.14) | 0.716 |
| Malignancy | 1.20 (0.53-2.76) | 0.654 |
| Eccentric × malignancy | 5.25(1.03-32.31) | 0.054 |

*Binary logistic regression model: 90-day recurrence as the dependent variable; morphology (eccentric vs. circumferential), malignancy (yes vs. no), and their interaction term were entered simultaneously. The interaction term was used to assess whether the association between morphology and recurrence differed by etiology.

**Supplementary Table 12. Interaction model for drainage strategy**

| **Variable in interaction model*** | **OR (95% CI)** | ***p-*value** |
| --- | --- | --- |
| Eccentric morphology | 1.2 (0.28-5.21) | 0.803 |
| Drainage strategy | 0.61 (0.17-2.26) | 0.443 |
| Eccentric × drainage strategy | 1.63 (0.28-9.32) | 0.576 |

*Binary logistic regression model: 90-day recurrence as the dependent variable; morphology (eccentric vs. circumferential), drainage strategy (TTE-guided vs. non-TTE-guided invasive treatment), and their interaction term were entered simultaneously. The interaction term was used to assess whether the association between morphology and recurrence differed by drainage strategy.

Supplementary Table 13. Baseline characteristics before and after 1:1 propensity score matching among patients discharged alive

| Variable | Before matching (n=147) | |  | After matching (n=84, 1:1) | |  |  |
| --- | --- | --- | --- | --- | --- | --- | --- |
|  | Circumferential  (n=103) | Eccentric  (n=44) | *p*-value | Circumferential  (n=42) | Eccentric  (n=42) | SMD | *p*-value |
| Age | 63.60 ±13.39 | 59.34 ±16.11 | 0.140 | 62.38 ±14.53 | 59.83 ±15.21 | 0.17 | 0.435 |
| Male | 55 (53.40%) | 22 (50.00%) | 0.706 | 21 (50.0%) | 22 (52.4%) | 0.05 | 1.000 |
| Complicated with pneumonia | 54 (52.43%) | 16 (36.36%) | 0.074 | 15 (35.7%) | 16 (38.1%) | 0.05 | 1.000 |
| Complicated with malignancy | 59 (57.28%) | 15 (34.09%) | 0.010* | 13 (31.0%) | 15 (35.7%) | 0.10 | 0.817 |
| CKD | 25 (24.27%) | 10 (22.73%) | 0.840 | 8 (19.0%) | 10 (23.8%) | 0.07 | 0.791 |
| Heart failure | 18 (17.48%) | 10 (22.73%) | 0.458 | 9 (21.4%) | 10 (23.8%) | 0.06 | 1.000 |

**Supplementary Table 14. Comparison of 90-day outcomes after 1:1 propensity score matching among patients discharged alive**

| Outcome | Before matching (n=147) | | |  | After matching (n=84, 1:1) | |  |
| --- | --- | --- | --- | --- | --- | --- | --- |
|  | Circumferential  (n=103) | Eccentric  (n=44) | | *p*-value | Circumferential  (n=42) | Eccentric  (n=42) | *p*-value |
| Effusion recurrence, n (%) | 36 (34.95%) | | 22 (50.00%) | 0.127 | 15 (35.6%) | 21 (50.0%) | 0.270 |
| Readmission, n (%) | 28 (27.18%) | | 16 (36.36%) | 0.360 | 12 (28.6%) | 15 (35.7%) | 0.641 |
| Repeat puncture, n (%) | 13 (12.62%) | | 5 (11.36%) | >0.999 | 5 (11.9%) | 4 (9.5%) | 1.000 |
| 90-day Death, n (%) | 6 (5.83%) | | 4 (9.09%) | 0.487 | 1 (2.4%) | 4 (9.5%) | 0.360 |
